# Safety and effectiveness of oral anticoagulants in patients with atrial fibrillation and stage 4 chronic kidney disease: a real-world experience

**DOI:** 10.1007/s11739-024-03658-9

**Published:** 2024-06-28

**Authors:** Rosa Talerico, Elisa Brando, Lorenzo Luzi, Maria Cristina Vedovati, Michela Giustozzi, Melina Verso, Leonardo Di Gennaro, Maria Basso, Antonietta Ferretti, Angelo Porfidia, Erica De Candia, Roberto Pola, Giancarlo Agnelli, Cecilia Becattini

**Affiliations:** 1 Department of Geriatric, Orthopedic, and Rheumatologic Sciences, Fondazione Policlinico Universitario A. Gemelli IRCCS, Università Cattolica del Sacro Cuore, Largo Francesco Vito, 1-00168 Rome, Italy; 2 IRCCS San Raffaele, Rome, Italy; 3 Diagnostic and Therapeutic Medicine Department, University Campus Bio-Medico of Rome, Rome, Italy; 4 Internal and Cardiovascular Medicine—Stroke Unit, University of Perugia, Perugia, Italy; 5 Department of Diagnostic Imaging, Radiotherapy, Oncology and Hematology, Hemorrhagic and Thrombotic Diseases Center, Fondazione Policlinico Universitario A. Gemelli IRCCS, Rome, Italy; 6 Department of Translational Medicine and Surgery, Università Cattolica del Sacro Cuore, Rome, Italy

**Keywords:** Non-valvular atrial fibrillation, Severe renal failure, Stage 4 chronic kidney disease, Oral anticoagulants, Vitamin K antagonists, Direct oral anticoagulants

## Abstract

It is still uncertain whether direct oral anticoagulants (DOACs) perform better than vitamin K antagonists (VKAs) in subjects with non-valvular atrial fibrillation (NVAF) and advanced chronic kidney disease (CKD). The aim of the study was to compare safety and effectiveness of DOACs and VKAs in patients with NVAF and stage 4 CKD (creatinine clearance 15–29 mL/min). We searched the hospital databases of two academic centers to retrospectively identify patients with stage 4 CKD who were on treatment with DOACs or VKAs for NVAF. Safety was the primary outcome of the study and was assessed in terms of incidence of major bleeding (MB). Secondary outcomes were clinically relevant non-major bleeding (CRNMB) and death for any cause. A total of 176 patients (102 on DOACs and 74 on VKAs) were found and included in the analysis. The incidence rate of MB was not statistically different between groups (8.6 per 100 patients-year in the DOAC group and 5.6 per 100 patients-year in the VKA group). Rates of IS/SSE and CRNMB were statistically similar in the two treatment groups, as well. There were less deaths for any cause in the DOAC group than in the VKA group (8.6 and 15.8 per 100 patients-year, respectively), but the difference was not statistically significant. This study found no difference in terms of safety and effectiveness between patients with NVAF and stage 4 CKD treated with DOACs and VKAs. Larger prospective or randomized studies are needed to confirm these findings.

**Supplementary Information:**

The online version contains supplementary material available at 10.1007/s11739-024-03658-9.

## Introduction

Nonvalvular atrial fibrillation (NVAF) is the most common arrhythmia of clinical significance and is associated with increased morbidity and mortality. It is estimated that 5 million new cases of NVAF occur each year worldwide [1]. The prevalence of NVAF increases with frailty and aging; thus, it is a frequent condition in the elderly population.

Chronic kidney disease (CKD), defined and classified according to the KDIGO nomenclature [2], is also a widespread condition affecting about 11–13% of the global population [3]. Importantly, up to 20% of patients with CKD also have NVAF. In patients with NVAF, the concomitant presence of CKD increases the risk of thrombotic events and the rate of morbidity and mortality from cardiovascular and cerebrovascular diseases. On the other hand, in subjects with NVAF, CKD also increases hemorrhagic risk [4]. The result is that anticoagulant therapy is particularly challenging in this subset of patients.

Currently, first choice drugs to prevent ischemic stroke (IS) or systemic embolism (SSE) in patients with NVAF are direct oral anticoagulants (DOACs). These drugs are either non-inferior or superior to vitamin K antagonists (VKAs) in preventing thrombotic events, with the advantage of a decreased intracranial bleeding risk [5]. However, the pivotal randomized clinical trials (RCTs) that have compared DOACs to VKAs in NVAF patients have excluded subjects with a creatinine clearance (CrCl) lower than 30 mL/min [6–8], with the only exception of a few patients with CrCl between 25 and 30 mL/min included in the ARISTOTLE trial [9]. Therefore, it is still uncertain whether DOACs perform better than VKAs, in terms of both safety and efficacy, in patients with severe CKD [10].

Based on this, we carried out a retrospective study to compare safety and effectiveness of DOACs and VKAs in real-life patients affected by both NVAF and severe CKD (CrCl 15–29 mL/min).

## Aim of the study

The aim of the study was to compare safety and effectiveness of DOACs and VKAs in a cohort of patient with NVAF and severe CKD (CrCl 15–29 mL/min).

## Methods

### Study design and population

This was a retrospective cohort study, conducted by searching the hospital databases of the University Hospital of Perugia and the Fondazione Policlinico Universitario A. Gemelli IRCCS of Rome, Italy. The search was limited to the period between 01 March 2013 and 31 March 2022. We searched for patients who were on anticoagulant therapy with DOACs or VKAs for NVAF and had severe CKD, which was defined as creatinine clearance between 15 and 29 mL/min and also labeled in the text as stage 4 CKD, calculated using either the Cockcroft–Gault (CG) or the Modification of Diet in Renal Disease (MDRD) formula. Exclusion criteria from the study were age < 18 years, valvular AF, use of other anticoagulants rather than DOACs and VKAs, and other indications to anticoagulant therapy rather than NVAF. Demographic, clinical, and laboratory data were collected for all patients, including comorbidities, HAS-BLED score [11], and CHA_2_DS_2_VASc score [12].

### Study outcomes

The primary outcome was major bleeding (MB), which was defined according to the criteria of the International Society of Thrombosis and Hemostasis [13] as fatal bleeding, and/or symptomatic bleeding in a critical area or organ (such as intracranial, intraspinal, intraocular, retroperitoneal, intra-articular or pericardial, or intramuscular with compartment syndrome), and/or bleeding causing a fall in hemoglobin level of 20 g L^−1^ (1.24 mmol L^−1^) or more, or leading to transfusion of two or more units of whole blood or red cells.

The secondary study outcomes were objectively confirmed ischemic stroke or systemic embolism, clinically relevant non-major bleeding (CRNMB), and all-cause death during anticoagulant treatment. CRNMBs were defined according to the ISTH criteria [14] as hemorrhages that did not fit the criteria for the definition of MB, but required medical intervention by a healthcare professional, or led to hospitalization or increased level of care, or prompted a face to face (i.e., not just a telephone or electronic communication) evaluation. Primary and secondary outcomes were extrapolated by the analysis of the hospital databases and further confirmed through review of individual medical records by at least two independent investigators in each participating center. Since we included both patients who already had stage 4 CKD at the time of DOAC or VKA prescription and patients who developed severe CKD stage 4 CKD when already on anticoagulant treatment, we only considered the events that occurred when stage 4 CKD was present.

### Statistical analysis

The clinical and demographic characteristics of the study population were reported as either percentages (for categorical variables) or mean ± standard deviation (SD) or median (interquartile range) for continuous variables. Student’s t test was used to compare continuous variables. Chi-squared test was used to compare categorical variables. The incidence of MB, SSE, CRNMB, and all-cause death were reported as rate per 100 patients-year. The cumulative rates of MBs were estimated using the Kaplan–Meier method and compared for DOACs and VKAs with the log-rank test. A multivariate Cox proportional hazards model was used to assess independent predictors of MBs. The considered variables were DOAC use (vs VKA use), age ≥ 75 years, HAS-BLED score as ordinal variable, hemoglobin value, and platelet count as continuous variables. Results were reported as hazard ratios (HR); 95% confidence intervals (CI) and *p* values were also shown. To explain any possible difference in the incidence of MB between the DOAC and VKA groups, demographic, laboratory, and clinical characteristics were reported using descriptive statistical techniques. All reported *p* values were two-sided, and *p* values below 0.05 were considered statistically significant. All statistical analyses were performed using SPSS software, version 27 (IBM Corporation, Armonk, NY).

## Results

### Baseline characteristics of the population

Our database search led to the identification of 176 patients. Of these, 102 were on DOAC and 74 on VKA therapy. The baseline characteristics of the study population are summarized in Table 1. No significant differences were observed in either median age or age ≥ 75 years between the two groups. The proportion of patients who already had stage 4 CKD when they were prescribed anticoagulant therapy was also similar in the DOAC and VKA groups. Congestive heart failure, diabetes, and peripheral vascular disease were significantly more prevalent in the VKA group, as well as the use of other medications with a possible impact on bleeding, i.e., aspirin, clopidogrel, and non-steroidal anti-inflammatory drugs (NSAIDs). Regarding laboratory tests, significantly lower CrCl and higher creatinine values were noticed in patients receiving VKAs in comparison to patients receiving DOACs. No significant differences were observed in the median HAS-BLED and CHA_2_DS_2_VASc scores between the two groups. Apixaban was the most frequently prescribed DOAC, followed by rivaroxaban, edoxaban, and dabigatran. In the DOAC group, there were 15 patients who were receiving an inappropriate treatment with dabigatran (which is not approved for the treatment of patients with stage 4 CKD) or with full doses of Factor Xa inhibitors (which should not be used in subjects with stage 4 CKD).

**Table 1:** Baseline characteristics of the study population

Table 1 Baseline characteristics of the study population			
	DOAC (*n* = 102)	VKA (*n* = 74)	*p* value
Demographic			
Age (years), median and interquartile range	87 (89–81)	83 (89–73.5)	0.11
≥ 75, *n* (%)	96 (94.1%)	69 (93.2%)	0.81
Range	57–101	66–98	NA
Female gender, *n* (%)	57 (55.9%)	44 (59.5%)	0.64
Laboratory tests			
Hemoglobin (g/dL), mean ± SD	11.9 ± 1.7	11.6 ± 1.6	0.14
Platelet (1,000/mm^3^), mean ± SD	208 ± 74	215 ± 96	0.58
Creatinine clearance, mean ± SD	26.4 ± 3.8	24.3 ± 4.7	**0.002**
Creatinine (mg/dL), mean ± SD	1.7 ± 0,6	2.2 ± 0.7	** < 0.001**
Clinical characteristics			
Congestive heart failure, *n* (%)	54 (52.9%)	59 (79.7%)	** < 0.001**
Hypertension, *n* (%)	97 (95.1%)	68 (91.9%)	0.24
Diabetes, *n* (%)	24 (23.5%)	29 (39.2%)	**0.03**
Previous stroke/TIA, *n* (%)	20 (19.6%)	15 (20.3%)	0.91
Vascular diseases, *n* (%)			
History of MI/angina	26 (25.5%)	19 (25.7%)	0.98
Peripheral artery disease	13 (12.7%)	18 (24.3%)	**0.05**
Liver disease, *n* (%)	4 (3.9%)	2 (2.7%)	0.66
Previous bleeding or predisposition, *n* (%)	12 (11.8%)	6 (8.1%)	0.33
Medication use predisposing to bleeding, *n* (%)	8 (7.8%)	12 (16.2%)	** < 0.001**
Alcohol use, *n* (%)	0 (0%)	0 (0%)	NA
HAS-BLED score (%)			
0–1	2.9%	4.0%	
2–3	80.4%	66.2%	
4–5	16.6%	28.4%	
> 6	2%	1.4%	
HAS-BLED score, median and interquartile range	3 (2–3)	3 (2–4)	0.06
CHA_2_DS_2_VASc score (%)			
0–1	1%	0%	
2–3	14.7%	5.4%	
4	22.5%	16.2%	
5–6	51.0%	56.8%	
7–9	10.8%	21.6%	
CHA_2_DS_2_VASc score, median and interquartile range	5 (4–6)	5 (4–6)	0.09
Naïve for anticoagulant therapy, *n* (%)	72 (70.6%)	56 (75.7%)	0.45
Duration of observation (*months*), mean ± SD	23.1 ± 17.1	31.7 ± 38,9	0.08
DOAC, *n* (%)			
Apixaban 5 mg BID	4 (3.9%)	NA	NA
Apixaban 2.5 mg BID	49 (48%)	NA	NA
Dabigatran 150 mg BID	0 (0%)	NA	NA
Dabigatran 110 mg BID	9 (8.8%)	NA	NA
Edoxaban 60 mg OD	1 (1%)	NA	NA
Edoxaban 30 mg OD	16 (15.7%)	NA	NA
Rivaroxaban 20 mg OD	1 (1%)	NA	NA
Rivaroxaban 15 mg OD	22 (21.6%)	NA	NA

Values in bold are those considered statistically significant (<0.05)

Values are presented as number (%), or mean ± standard deviation

^*^Aspirin, clopidogrel, NSAIDs

*BID* twice a day, *OD* once a day, *NA* not available

### Clinical outcomes in the DOAC and VKA groups

**Fig. 1:**
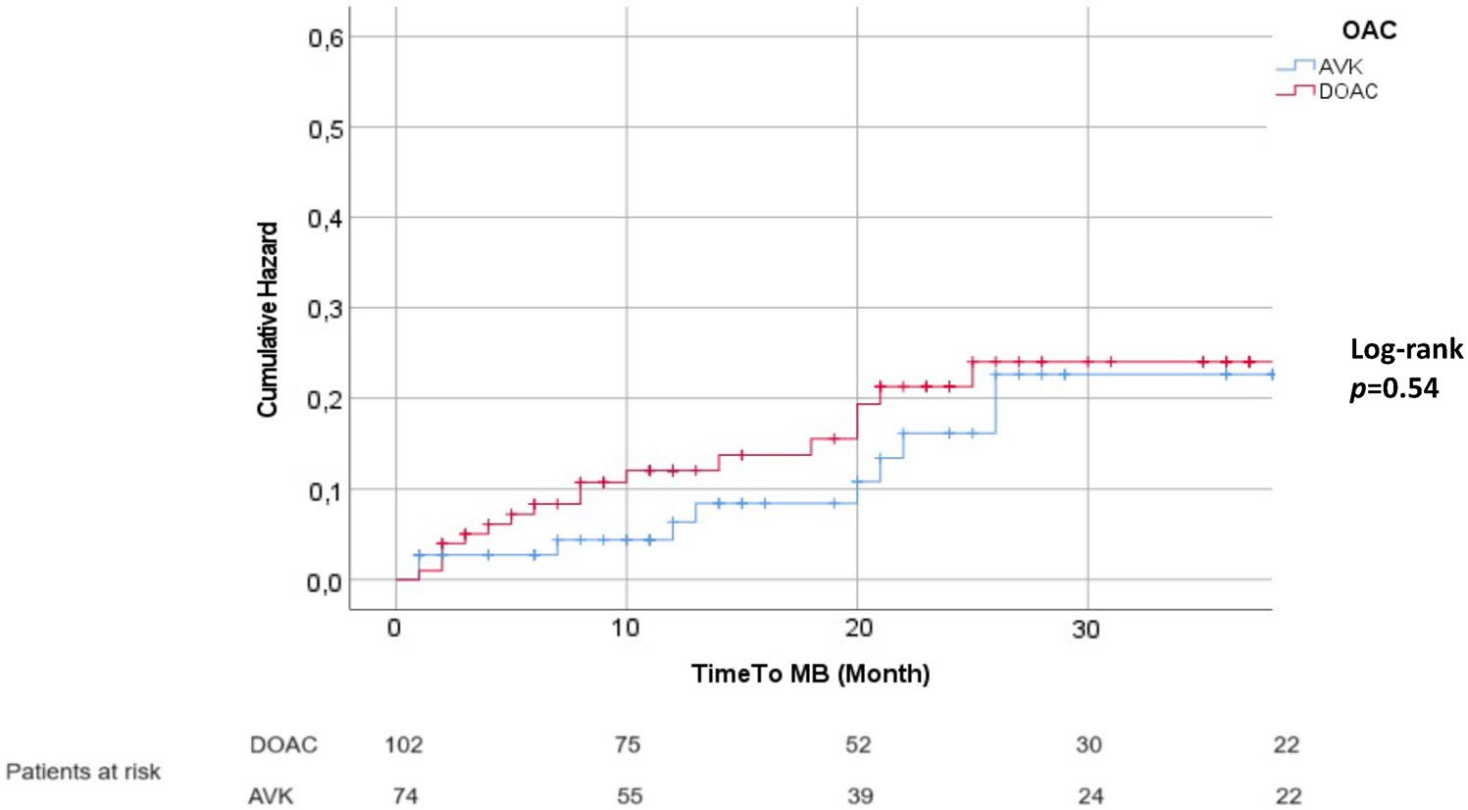
Incidence of MBs between DOAC and VKA groups. *OAC(s*) oral anticoagulants, *VKA(s)* vitamin K antagonists, *DOAC(s)* direct oral anticoagulants, *CI* confidence interval, *HR* hazard ratio

A total of 28 MBs were detected in our study population. Of these, 17 were in the DOAC group and 11 in the VKA group. The total observation period was of 197 patients-year in the DOAC group and 196 patients-year in the VKA group. Thus, the incidence rate of MB in the DOAC group was 8.6 per 100 patients-year, while in the VKA group was 5.6 per 100 patients-year (Table 2). The cumulative rates of MB events in patients receiving DOACs or VKAs are reported in Fig. 1. The type and site of MBs are detailed in Table 2.

**Table 2:** Study outcome events

Table 2 Study outcome events	No. of patients (100 patients-year)	
	DOAC (*n* = 102)	VKA (*n* = 74)
Primary study outcome		
Major bleeding (MB)	17 (8.6)	11 (5.6)
Type of MB		
Fatal bleeding	1 (0.5)	2 (1.0)
Fall in Hb level of 2 g/dL or transfusion (2 or more U)	11 (5.6)	3 (1.5)
Symptomatic bleeding in a critical area	5 (2.5)	6 (3.0)
Site of MB		
Gastrointestinal (GI)	7 (3.6)	4 (2.0)
Intracerebral hemorrhage (ICH)	3 (1.5)	4 (2.0)
Others	7 (3.6)	3 (1.5)
Secondary study outcomes		
Ischemic stroke or systemic embolism	2 (1.0)	0 (0.0)
Clinically relevant non-major bleeding	5 (2.5)	6 (3.0)
Death	17 (8.6)	31 (15.8)

As mentioned above, in the DOAC group, there were 15 patients who were receiving an inappropriate anticoagulant treatment, either in terms of type of drug (dabigatran) or drug dosage (full doses of Factor Xa inhibitors). Among these 15 patients, 5 presented MBs. In this subgroup, the total observation period was 29 patients-year, and the incidence rate of MB was 17.5 per 100 patients-year. On the other hand, among the 87 DOAC patients who were properly treated (reduced doses of Factor Xa inhibitors), 12 presented MBs. In this subgroup of patients, the total observation period was 168 patients-year, and the incidence rate of MB was 7.1 per 100 patients-year.

Regarding secondary outcomes, we detected 2 ischemic strokes, 11 CRNMBs, and 48 deaths. The 2 ischemic events were observed in the DOAC group, with an incidence rate of 1.0 per 100 patients-year. There were no ischemic events detected in the VKA group. Of the 11 CRNMBs, 5 occurred in the DOAC group, with an incidence rate of 2.5 per 100 patients-year, and 6 in the VKA group, with an incidence rate of 3.0 per 100 patients-year. Of the 48 deaths for any cause, there were 17 and 31 in the DOAC and VKA group, respectively, with an incidence rate of 8.6 and 15.8 per 100 patients-year, respectively.

Patients with (*n* = 28) and without (*n* = 148) MBs only differed for baseline hemoglobin levels (which were higher in patients without MBs) (Table S1). Of the 148 total patients who did not suffer MBs, 85 were on treatment with DOACs and 63 with VKAs. Patients on VKAs presented lower CrCl and higher creatinine values (Table S2). Heart failure, diabetes, and medications with a potential impact on bleeding were more frequent in the VKA group.

Finally, we report a description of the main characteristics of the patients who had MBs (*n* = 28 in total, *n* = 17 in the DOAC group and *n* = 11 in the VKA group). Patients in the VKA group had significantly higher creatinine values and significantly lower CrCl (Table S3).

## Predictive factors associated with the development of a major bleeding event

At Cox proportional hazards regression analysis, an increase in HAS-BLED score (HR 1.59; 95% CI 1.09–2.30) and a decrease in hemoglobin values (HR 0.71; 95% CI 0.57–0.90) were significantly associated with MB, while DOAC use (versus VKA) was not (HR 1.34; 95% CI 0.58–3.08). Complete results are reported in Table 3.

**Table 3:** Results of Cox proportional hazards regression analysis

Table 3 Cox proportional hazards model for major bleeding events		
Variable	HR (95% CI)	*p* value
DOAC (vs*. VKA*)	1.34 (0.58–3.08)	0.49
Age ≥ 75	1.43 (0.19–10.82)	0.73
Has-bled	1.59 (1.09–2.30)	**0.016**
Hemoglobin	0.71 (0.57–0.90)	**0.004**
Creatinine clearance	1.06 (0.96–1.18)	0.23

Values in bold are those considered statistically significant (<0.05)

*VKA* vitamin K antagonists, *DOAC* direct oral anticoagulants, *CI* confidence interval, *HR* hazard ratio

## Discussion

It is still uncertain whether DOACs are better than VKAs in subjects with advanced CKD. The reason is that RCTs have not included this type of patients and the evidence available in the literature is only based on meta-analyses and observational studies or registries. Not surprisingly, the result is that data in the literature are too heterogeneous to draw firm conclusions. For instance, there is a metanalysis [15] including subgroup data from RCTs (ARISTOTLE trial [9]) and observational studies that has shown that, in patients with advanced CKD (CrCl < 30 mL/min) and NVAF, DOACs significantly reduced the risk of SSE [pooled HR 0.60; 95% CI, 0.43 to 0.85), *I*^2^ = 0.0%] and MB [pooled HR 0.74; 95% CI, 0.59 to 0.93), *I*^2^ = 30.4%] compared to warfarin. However, the authors clearly state the several limitations of a study-level meta-analysis, as it was not possible to consider confounders at the individual patient level and the heterogeneity was significant, so interpretation of the considered events requires attention.

Recent observational studies are also available. Hsu et al. [16] found that DOACs were associated with a lower risk of ischemic events compared with warfarin in patients with AF and advanced CKD (CrCl < 30 mL/min), and, among DOACs, apixaban was linked to a substantial reduction in the risk of ischemia and hemorrhage compared with warfarin. Anyway, in these as in other studies, the limitations of are not negligible and the data must be critically analyzed.

In our retrospective cohort, we found that DOACs and VKAs were associated with similar rates of MB, CRNMB, SSE, and all-cause mortality. Therefore, the use of DOACs was not associated with increased risk of bleeding in that in patients with NVAF and stage 4 CKD. The only parameters associated with MB, in the whole population, were the HAS-BLED score and decreased hemoglobin values.

Analyzing our results, an important point to underline is that 15 patients in our cohort were receiving an inappropriate anticoagulant treatment. This is of note because 5 of the registered MBs occurred in this subgroup of patients. This finding strengthens the concept that inappropriate use of anticoagulant medications increases the risk of bleeding, and this is particularly true in subjects with impaired renal function [17]. Indeed, a recent individual patient-level network meta-analysis [18] evaluated the safety and efficacy of DOACs versus warfarin based on continuous CrCl. In this meta-analysis, in patients with the worst kidney function (down to a CrCl of 25 mL/min), standard-dose DOACs were safer and more effective than warfarin and lower-dose DOACs did not significantly lower the incidence of bleeding or ICH compared with standard-dose DOACs, but were associated with a higher incidence of stroke/systemic embolism and death. The authors conclude that inappropriate dose reduction of DOACs likely results in a higher risk of thromboembolism and death without reducing the risk of bleeding or intracranial hemorrhage.

By performing a detailed analysis of MBs, it is possible to see that there was no difference between the DOAC and VKA groups in terms of anatomical sites of bleedings either. Indeed, we observed 7 gastrointestinal bleedings (GIB) and 3 intracranial hemorrhages (ICH) in the DOAC group (3.6 and 1.5 per 100 patients-year, respectively), and 4 GIB and 4 ICH in the VKA group (2.0 per 100 patients-year).

Additional considerations should be made on the mortality rate observed in our cohort. First, there were 48 all-cause deaths, which means that almost 30% of the study population died during the time considered by our analysis. This is consistent with the notion that subjects with severe CKD and NVAF are extremely fragile and have reduced life expectancy. Second, mortality rate was higher in the VKA than in the DOAC group (15.8 vs. 8.6 per 100 patients-year, respectively), although the difference was not statistically significance. Such finding could be due, at least in part, to the higher disease burden displayed by patients in the VKA group. Indeed, mean CHA_2_DS_2_VASc and HAS-BLED scores were higher among patients treated with VKA than those treated with DOACs. In addition, patients in the VKA group had significantly higher creatinine levels and a greater use other drugs with a potential impact on bleeding (such as antiplatelet and NSAID). Taken together, these considerations suggest that death might have been a competitive event compared with the occurrence of the other outcomes. It is also possible that, in real-life, there may still be a tendency to prescribe VKAs to frailer patients.

An important question to address when considering anticoagulation for patients with NVAF and advanced CKD is whether or not the benefit of stroke prevention outweighs the risk of bleeding. Patients with advanced CKD and NVAF constitute a high-risk population characterized by increased hemorrhagic and ischemic risk, which may impact the net clinical benefit associated with anticoagulant therapy. As reported in a large Dutch cohort study [19], a CrCl of < 45 mL min^−1^ 1.73 m^−2^ with albuminuria (stage G3b-5A2/3 chronic kidney disease) was associated with a 3.5-fold increased risk of bleeding (95% CI 2.3–5.3) compared with patients without CKD.

In this cohort, we found a high number of MBs (cumulative incidence of 15.9%) and a small number of ischemic events (cumulative incidence 1.1%). This finding expresses and confirms the efficacy profile of oral anticoagulants, but raises important questions about safety. Indeed, in a 2017 study by Cho et al. [20], it was shown that moderate to severe renal impairment in NVAF patients increased bleeding risk regardless of antithrombotic treatment, while SSE risk increased only in patients not receiving antithrombotic treatment during follow-up.

This study has several strengths. First, it is a real-life study that has included a well-selected category of patients with a homogeneous indication to oral anticoagulation. Second, the study has a long follow-up period. Third, the study cohort mainly consists of greatly elderly patients. Finally, it is a multicenter study.

The study also has limitations. One is the retrospective nature of the analysis. Due to that, it is possible that some outcome events were missed. Another limitation is that it was not always possible to calculate the CrCl using the CG formula, due to the lack of exact body weight for some patients. When this was the case, the MDRD formula was used. This formula might overestimate renal function [21]. In addition, it was not always possible to trace the PT-INR value of all the patients in the VKA group. This is the reason why time in therapeutic range (TTR) has not been reported. Lastly, comparisons of the bleeding risk among different types of DOAC drugs were not evaluated due to the small number of patients analyzed. Furthermore, although apixaban was the most prescribed DOAC in our population, a result in line with the latest available evidence [22], also shown in studies of venous thromboembolism patients with CKD [23], the sample size was not large enough to express firm judgments about one drug over another in this category.

## Conclusions

Patients with advanced stage 4 CKD and NVAF have a considerable risk of bleeding events during oral anticoagulant treatment. Our retrospective analysis, performed in two Italian academic hospitals, did not find a statistically significant difference between patients treated with DOACs and VKAs in terms of both safety and effectiveness. There was a higher number of MBs among patients treated with the inappropriate type of DOAC or an inappropriate dose of DOAC. The rate of all-cause death was lower in the DOAC than the VKA group, but this difference was not statistically significant. Randomized controlled trials and/or prospective studies on larger populations are needed to confirm these findings.

## Supplementary Information

Below is the link to the electronic supplementary material.

Supplementary file1 (DOCX 35 KB)

## Data Availability

Not applicable.
