# Supplementary material for: Safety and effectiveness of oral anticoagulants in patients with atrial fibrillation and stage 4 chronic kidney disease: a real-world experience
Source: Intern Emerg Med. 2024 Jun 28;19(6):1645–52. doi: 10.1007/s11739-024-03658-9 (PMC11405423; doi:10.1007/s11739-024-03658-9)
Supplement: Supplementary file 1 — Supplementary file1 (DOCX 35 KB) [file 11739_2024_3658_MOESM1_ESM.docx]

**Safety and effectiveness of oral anticoagulants in patients with atrial fibrillation and four stage renal disease: a real-world experience.**

**Authors:** Rosa Talerico^1-2^, Elisa Brando^3^, Lorenzo Luzi^4^, Maria Cristina Vedovati^4^, Michela Giustozzi^4^, Melina Verso^4^, Leonardo Di Gennaro^5^, Maria Basso^5^, Antonietta Ferretti^5^, Angelo Porfidia^1^, Erica De Candia^6^, Roberto Pola^1^, Giancarlo Agnelli^4^, Cecilia Becattini^4^

**Affiliation:**

*^1^Department of Geriatric, Orthopedic, and Rheumatologic Sciences, Fondazione Policlinico Universitario A. Gemelli IRCCS, Università Cattolica del Sacro Cuore, Rome, Italy.*

*^2^IRCCS San Raffaele, Rome, Italy.*

*^3^Diagnostic and Therapeutic Medicine Department, University Campus Bio-Medico of Rome, Rome, Italy.*

*^4^Internal and Cardiovascular Medicine – Stroke Unit, University of Perugia, Perugia, Italy.*

*^5^Department of Diagnostic Imaging, Radiotherapy, Oncology and Hematology, Hemorrhagic and Thrombotic Diseases Center, Fondazione Policlinico Universitario A. Gemelli IRCCS, Rome, Italy.*

*^6^Department of Traslational Medicine and Surgery, Università Cattolica del Sacro Cuore, Rome, Italy.*

**Corresponding Author:**

Dr. Rosa Talerico

[rosa.talerico@unicatt.it](mailto:rosa.talerico@unicatt.it)

L.go Francesco Vito, 1 – 00168 Rome, Italy (IT)

| **Table S1. Baseline characteristics of the overall study population without and with MBs** | | | |
| --- | --- | --- | --- |
|  | **Without MBs (N=148)** | **With MBs (N=28)** | ***p* value** |
| ***Demographic*** | | | |
| **Age (years), median and interquartile range** | 86 (89-80) | 86 (88-81) | 0.92 |
| ≥ 75, *n* (%) | 138 (93.2%) | 27 (96.4%) | 0.52 |
| Range | 57-101 | 72-93 | NA |
| **Female gender, *n* (%)** | 85 (57.4%) | 16 (57.1%) | 0.98 |
| ***Laboratory tests*** | | | |
| **Hemoglobin (g/dL), mean ± SD** | 11.9 ± 1.6 | 11.0 ± 1.6 | **0.006** |
| **Platelet (1,000/mm3), mean ± SD** | 207 ± 79 | 228 ± 104 | 0.32 |
| **Creatinine clearance, mean ± SD** | 25.4 ± 4.3 | 26.2 ± 4.5 | 0.36 |
| **Creatinine (mg/dL), mean ± SD** | 1.9 ± 0.7 | 1.73 ± 0.58 | 0.18 |
| ***Clinical characteristics*** | | | |
| **Congestive heart failure, *n* (%)** | 94 (65.3%) | 19 (67.9%) | 0.8 |
| **Hypertension, *n* (%)** | 138 (93.9%) | 27 (96.4%) | 0.6 |
| **Diabetes, *n* (%)** | 46 (31.1%) | 7 (25%) | 0.52 |
| **Previous stroke/TIA, *n* (%)** | 28 (18.9%) | 7 (25%) | 0.46 |
| **Vascular diseases, *n* (%)** | | | |
| History of MI/angina | 39 (26.4%) | 6 (21.4%) | 0.59 |
| Peripheral artery disease | 24 (16.2%) | 7 (25%) | 0.27 |
| **Liver disease, *n* (%)** | 6 (4.1%) | 0 (0%) | 0.29 |
| **Previous bleeding or predisposition*, *n* (%)** | 16 (10.8%) | 3 (10.7%) | 0.99 |
| **Medication use predisposing to bleeding, *n* (%)** | 32 (21.8%) | 5 (18.5%) | 0.7 |
| **Alcohol use, *n* (%)** | 0 (0.0%) | 0 (0.0%) | NA |
| **HAS-BLED score, *n* (%)** |  | | |
| 0-1 | 5 (3.4%) | 1 (3.6%) |  |
| 2-3 | 112 (75.7%) | 19 (67.8%) |  |
| 4-5 | 31 (20.9%) | 7 (25%) |  |
| >6 | 0 (0%) | 1 (3.6%) |  |
| **HAS-BLED score, median and interquartile range** | 3 (3-2) | 3 (4-2.25) | 0.52 |
| **CHA_2_DS_2_-VASc score, *n* (%)** |  | | |
| 0–1 | 1 (0.7%) | 0 (0.0%) |  |
| 2–3 | 14 (9.5%) | 5 (17.9%) |  |
| 4 | 29 (19.6%) | 6 (21.4%) |  |
| 5–6 | 79 (32.4%) | 15 (43.6%) |  |
| 7–9 | 25 (16.9%) | 2 (7.2%) |  |
| **CHA_2_DS_2_-VASc score, median and interquartile range** | 5 (6-4) | 5 (5.75-4) | 0.26 |
| **Naïve for anticoagulant therapy, *n* (%)** | 111 (75.0%) | 17 (60.7%) | 0.12 |
| **Duration of observation (months), mean ± SD** | 29.1 ± 29.9 | 14.1 ± 15.2 | **0.02** |

***Table S1. Baseline characteristics of the overall study population without and with MBs.***

**Aspirin, clopidogrel, NSAIDs.*

*Abbreviations. MBs: major bleedings.*

| **Table S2. Baseline characteristics of the overall study population without MBs** | | | |
| --- | --- | --- | --- |
|  | **On DOACs (N=85)** | **On VKAs (N=63)** | ***p* value** |
| ***Demographic*** | | | |
| **Age (years, median and interquartile range)** | 87 (89-81) | 83 (89-79) | 0.13 |
| ≥ 75, *n* (%) | 80 (94.1%) | 58 (92.1%) | 0.62 |
| Range | 57 - 101 | 66 - 98 | NA |
| **Female gender, *n* (%)** | 49 (57.6%) | 36 (57.1%) | 0.96 |
| ***Laboratory tests*** | | | |
| **Hemoglobin (g/dL), mean ± SD** | 12.1 ± 1.6 | 11.7 ± 1.6 | 0.11 |
| **Platelet (1,000/mm3), mean ± SD** | 206 ± 65 | 208 ± 94 | 0.89 |
| **Creatinine clearance, mean ± SD** | 26.1 ± 3.9 | 24.4 ± 4.7 | **0.02** |
| **Creatinine (mg/dL), mean ± SD** | 1.7 ± 0.6 | 2.23 ± 0.7 | **<0.001** |
| ***Clinical characteristics*** | | | |
| **Congestive heart failure, *n* (%)** | 43 (53.1%) | 51 (81%) | **<0.001** |
| **Hypertension, *n* (%)** | 81 (96.4%) | 59 (90.5%) | 0.14 |
| **Diabetes, *n* (%)** | 20 (23.5%) | 26 (41.3%) | **0.02** |
| **Previous stroke/TIA, *n* (%)** | 16 (18.8%) | 12 (19%) | 0.97 |
| **Vascular diseases, *n* (%)** | | | |
| History of MI/angina | 23 (27.1%) | 16 (25.4%) | 0.82 |
| Peripheral artery disease | 10 (11.8%) | 14 (22.2%) | 0.09 |
| **Liver disease, *n* (%)** | 4 (4.7%) | 2 (3.2%) | 0.64 |
| **Previous bleeding or predisposition*, *n* (%)** | 12 (14.1%) | 4 (6.3%) | 0.13 |
| **Medication use predisposing to bleeding, *n* (%)** | 25 (29.8) | 7 (11.1%) | **0.01** |
| **Alcohol use, *n* (%)** | 0 (0.0%) | 0 (0.0%) | NA |
| **HAS-BLED score, *n* (%)** |  |  | |
| 0-1 | 2 (2.4%) | 3 (4.8%) |  |
| 2-3 | 70 (82.3%) | 42 (66.7%) |  |
| 4-5 | 13 (15.3%) | 18 (28.5%) |  |
| >6 | 0 (0%) | 0 (0%) |  |
| **HAS-BLED score, median and interquartile range** | 5 (3-2) | 3 (4-2) | 0.08 |
| **CHA_2_DS_2_-VASc score, *n* (%)** |  |  | |
| 0–1 | 1 (1.2%) | 0 (0.0%) |  |
| 2–3 | 6 (7.1%) | 8 (12.7%) |  |
| 4 | 14 (16.5%) | 15 (23.8%) |  |
| 5–6 | 46 (54.1%) | 33 (23.8%) |  |
| 7–9 | 18 (21.2%) | 7 (11.2%) |  |
| **CHA_2_DS_2_-VASc score, median and interquartile range** | 5 (6-4.5) | 2 (6-4) | 0.1 |
| **Naïve for anticoagulant therapy, *n* (%)** | 64 (75.3%) | 47 (74.6%) | 0.92 |
| **Duration of observation (months), mean ± SD** | 25.8 ± 17.2 | 33.7 ± 41.1 | 0.16 |

***Table S2. Baseline characteristics of the overall study population without MBs.***

**Aspirin, clopidogrel, NSAIDs.*

*Abbreviations. DOACs: direct oral anticoagulants; VKAs: vitamin K antagonists.*

| **Table S3. Baseline characteristics of the overall study population with MBs** | | | |
| --- | --- | --- | --- |
|  | **On DOACs (N=17)** | **On VKAs (N=11)** | ***p* value** |
| ***Demographic*** | | | |
| **Age (years, median and interquartile range)** | 86 (89-81.50) | 84 (88-80) | 0.71 |
| ≥ 75, *n* (%) | 16 (94.1%) | 11 (100%) | 0.41 |
| Range | 72 - 92 | 76 - 93 | NA |
| **Female gender, *n* (%)** | 8 (47.1%) | 8 (72.7%) | 0.18 |
| ***Laboratory tests*** | | | |
| **Hemoglobin (g/dL), mean ± SD** | 11.1 ± 1.8 | 10.9 ± 1.4 | 0.79 |
| **Platelet (1,000/mm3), mean ± SD** | 213 ± 106 | 251 ± 100 | 0.35 |
| **Creatinine clearance, mean ± SD** | 27.8 ± 3.5 | 23.8 ± 5.0 | **0.04** |
| **Creatinine (mg/dL), mean ± SD** | 1.5 ± 0.5 | 2.1 ± 0.4 | **0.001** |
| ***Clinical characteristics*** | | | |
| **Congestive heart failure, *n* (%)** | 11 (64.7%) | 8 (72.7%) | 0.66 |
| **Hypertension, *n* (%)** | 16 (94.1%) | 11 (100%) | 0.41 |
| **Diabetes, *n* (%)** | 4 (23.5%) | 3 (27.3%) | 0.82 |
| **Previous stroke/TIA, *n* (%)** | 4 (23.5%) | 3 (27.3%) | 0.82 |
| **Vascular diseases, *n* (%)** | | | |
| History of MI/angina | 3 (17.6%) | 3 (27.3%) | 0.54 |
| Peripheral artery disease | 3 (17.6%) | 4 (36.4%) | 0.26 |
| **Liver disease, *n* (%)** | 0 (0%) | 0 (0%) | NA |
| **Previous bleeding or predisposition*, *n* (%)** | 1 (5.9%) | 2 (18.2%) | 0.30 |
| **Medication use predisposing to bleeding, *n* (%)** | 2 (12.5%) | 3 (27.3%) | 0.33 |
| **Alcohol use, *n* (%)** | 0 (0.0%) | 0 (0.0%) | NA |
| **HAS-BLED score, *n* (%)** |  |  | |
| 0-1 | 1 (5.9%) | 0 (0%) |  |
| 2-3 | 12 (70.6%) | 7 (63.6%) |  |
| 4-5 | 4 (23.6%) | 3 (27.3%) |  |
| >6 | 0 (0%) | 1 (9.1%) |  |
| **HAS-BLED score, median and interquartile range** | 5 (3.50-2) | 3 (5-3) | 0.67 |
| **CHA_2_DS_2_-VASc score, *n* (%)** |  |  | |
| 0–1 | 0 (0.0%) | 0 (0.0%) |  |
| 2–3 | 2 (11.8%) | 3 (27.3%) |  |
| 4 | 4 (23.5%) | 2 (18.2%) |  |
| 5–6 | 10 (58.8%) | 5 (18.2%) |  |
| 7–9 | 1 (5.9%) | 1 (9.1%) |  |
| **CHA_2_DS_2_-VASc score, median and interquartile range** | 5 (6-4) | 5 (5-3) | 0.67 |
| **Naïve for anticoagulant therapy, *n* (%)** | 8 (47.1%) | 9 (81.8%) | 0.07 |
| **Duration of observation (months), mean ± SD** | 9.9 ± 8.0 | 20.6 ± 21.1 | 0.07 |

***Table S3. Baseline characteristics of the overall study population with MBs.***

**Aspirin, clopidogrel, NSAIDs.*

*Abbreviations. DOACs: direct oral anticoagulants; VKAs: vitamin K antagonists.*
